# Opinions Concerning the Case of Joanna Southcott

**Published:** 1814-10

**Authors:** 


*For the Medical and Physical Journal.*

### *Opinions concerning the Case of Joanna Southcott.*

YOU will readily conceive our anxiety for news on our first return to old England; but, whilst we were waiting with eagerness for something about the Princess of Wales, or the Princess Charlotte, nothing resounded on our deck but that a boy had died with child undelivered, and that a virgin that of 65 was pregnant. Some of the most ingenious among us accounted for these wonders by concluding that all hands were at work to repair the ravages of the late war. To others the first part of the intelligence was so well known, as to direct the whole inquiry to the old lady's condition. Here it was said there could be no doubt, as several medical gentlemen had attested it. Among others, I heard the name of my old master, whose certificate of my regular attendance on his lectures I had often exhibited with pleasure, not to say with triumph. I soon found all eyes fixed on me. It was in vain to urge the impossibility of such an illusion. The words of Mrs. Southcott were the only answer I could hear. From jokes we seemed approaching to something more serious, when an old lieutenant, determined to see the fun out, begged they would not be so hard upon *their doctor*, but hear him out.

Delighted at this apparent candour, I now began to explain myself with much formality, and was pleased with the profound attention with which I fancied myself listened to. Every thing went on very well till I attempted to explain the probable cause of the sensation like *life within her*. On this subject I began by remarking, that, if the apparent pregnancy arose from tumours connected with the uterus or its appendages, the gradual increase of such tumours might produce a motion in the intestines, which the old woman might fancy the motion of the child; or even the contractile power of the tunics of hydatids, which many medical writers consider as animals, might produce a sensation which might be fairly applicable to Mrs. Southcott's condition. An universal peel of laughter succeeded, after which, every attempt at renewing the subject proved abortive, and the gentleman who had set me a-going thought it time to check me. With the kindest intentions in the world, he asked, whether I was sure that, whatever might have been the former merits of my master, he was not now in his dotage? A cannon-ball could scarcely have silenced me more effectually. Is such the lot of human nature, thought I,—such the change in one whose eloquence a few years ago could arrest the attention upon the most intricate subjects,—the man but for whom Mr. Hunter would have been known only by his discoveries in natural history, whilst his medical doctrines would have remained unknown, or not understood,—the man by whom a Baillie submitted to be taught on a subject connected with his own particular department.* Is such a man,

* See Dr. Baillie's candid note on the cancerous uterus, in his second and every subsequent edition of his Morbid Anatomy.

in in so short a time, reduced so low?—I suppose my countenance spoke for me. A dull silence succeeded the former peels of laughter, whilst I retired with precipitation.

I was much gratified with Dr. Adams's Treatise on Hereditary Diseases, a subject on which we had always heard him with peculiar pleasure, and expressed our wish that he would publish, as nothing resembling his doctrine was to be met with in any other writer. Dr. A., in my opinion, has successfully combated Mr. Locke's definition of madness; offered the first explanation of scrofula, a term to be met with every where, but no where explained; and corrected a most important error copied from Aretæus, by almost every writer on the subject of leprosy. This error, I find by your Journal, has been revived by a late writer in his compilation on Cutaneous Diseases, and is again confuted by referring to a case at this time accessible to all the faculty.

But I am probably telling what every one knows excepting ourselves, to whom every thing is interesting, because every thing is new.

*A Medical Officer in His Majesty's Service.*

*Portsmouth*, *Sept.* 6, 1814.

